# Recent Developments in Eating Disorders in Children: A Comprehensive Perspective

**DOI:** 10.3390/jcm14176042

**Published:** 2025-08-26

**Authors:** Silvia Cimino, Arturo Bevilacqua, Luca Cerniglia

**Affiliations:** 1 Department of Dynamic, Clinical and Health Psychology, Sapienza University of Rome, 00186 Rome, Italy; silvia.cimino@uniroma1.it (S.C.); arturo.bevilacqua@uniroma1.it (A.B.); 2 Faculty of Psychology, International Telematic University Uninettuno, 00186 Rome, Italy

**Keywords:** eating disorders, early-onset, child psychopathology, biopsychosocial model

## Abstract

Recent years have witnessed a concerning rise in early-onset eating disorders (EDs), prompting a re-evaluation of their etiology, diagnosis, and treatment within pediatric populations. This perspective article synthesizes emerging evidence on the multifactorial origins of EDs in children, emphasizing a biopsychosocial framework that integrates genetic, epigenetic, psychological, and environmental factors. While early manifestations often diverge from adolescent or adult profiles—marked by somatic complaints, selective eating, and ritualistic behaviors—the disorders significantly interfere with developmental milestones. The COVID-19 pandemic has further exacerbated vulnerabilities, acting as a catalyst for disordered eating behaviors through increased familial stress, isolation, and disrupted routines. Central to this framework is the role of parental psychopathology and parent–child feeding interactions, which profoundly shape children’s emotional regulation and attachment patterns. Recent studies also underscore genetic susceptibilities—especially variants in the DRD4 and DAT1 genes—and epigenetic modifications that may mediate the transmission of risk across generations. The article reviews evidence from observational and genomic studies, highlighting how altered gene expression linked to early environmental stress contributes to the heterogeneity of EDs. Finally, it evaluates prevention and intervention strategies, including family-based treatments, digital health tools, and school-based programs. These strategies are essential for timely detection, individualized care, and reducing long-term impairment. Overall, the paper advocates for a nuanced understanding of EDs in children—recognizing their complex origins and developmental implications—to inform clinical practice, public health policy, and future research in pediatric mental health.

## 1. Introduction

Eating disorders (EDs) are complex psychiatric conditions characterized by severe disturbances in eating behaviors, distorted body image, and an intense fear of gaining weight [1]. These disorders include anorexia nervosa, bulimia nervosa, binge eating disorder, and other specified feeding or eating disorders (OSFEDs), each with distinct diagnostic criteria and clinical features. While once thought to be rare in childhood, recent years have seen a striking increase in early-onset EDs, shifting the diagnostic and therapeutic landscape significantly. In fact, the age of onset for many EDs is decreasing, with cases now frequently identified in children under the age of 12. This trend raises significant developmental and clinical concerns, as childhood-onset EDs often present with more severe symptomatology, rapid weight loss, and higher rates of psychiatric comorbidity compared to adolescent or adult-onset EDs [2].

Children with EDs may exhibit different clinical profiles than adolescents, such as more prominent somatic complaints, selective eating, or ritualistic behaviors around food [3]. These manifestations are often entangled with other developmental processes, including cognitive immaturity, limited emotional vocabulary, and dependence on parental figures for food provision and emotional regulation. Consequently, these disorders can interfere with critical developmental milestones, such as identity formation, socialization, and emotional self-regulation, thereby increasing the risk of long-term psychological impairment if not adequately addressed [4].

In addition to emotional regulation and food provision, parents play a central role in shaping children’s eating behaviors through modeling, knowledge transmission, and daily interaction patterns [5]. Parental attitudes toward food, body image, and health significantly influence children’s relationship with eating and self-perception. A lack of coherent guidance and role modeling may hinder the development of healthy habits, increasing the risk of disordered eating. Moreover, the promotion of physical activity within the family context can act as a protective factor, reducing stress and enhancing children’s capacity to make healthier food choices, thereby contributing to a more resilient developmental trajectory [6].

Epidemiologically, multiple studies have shown a rise in ED prevalence among both boys and girls during the last two decades. Although girls remain disproportionately affected, recent research reveals increasing diagnoses among boys, particularly in the context of muscularity-oriented concerns and compulsive exercise patterns. This gender expansion challenges traditional assumptions and underscores the need for gender-sensitive assessment tools and interventions [5,6]. Moreover, the growing cultural emphasis on appearance, exacerbated by social media and digitally altered images, contributes to the normalization of disordered eating and distorted body ideals even among very young children.

A substantial body of research has documented the profound impact of the COVID-19 pandemic on children and adolescents’ mental health, emphasizing a notable increase in the prevalence of EDs, particularly within contexts of heightened anxiety, isolation, and disrupted routines [7,8]. The pandemic has been characterized as a powerful distressing factor, exacerbating existing vulnerabilities and presenting new psychosocial stressors, including disrupted schooling, social isolation, and reduced physical activity, all of which are associated with higher ED risk [9,10]. For instance, Cerniglia and Cimino (we) found a significant rise in ED symptoms in adolescents during the pandemic, alongside increased internalizing and externalizing psychopathological symptoms [11].

At the heart of these developments is a need to reexamine the etiological frameworks that shape our understanding of EDs. To fully appreciate the multifactorial origins of EDs in children, traditional psychological models—such as psychodynamic, cognitive-behavioral, and family systems theories—must now be integrated with advances in neuroscience, genetics, and epigenetics. The concept of multifinality, wherein a single risk factor (e.g., parental psychopathology or early trauma) can lead to diverse psychological outcomes, and equifinality, where different pathways (e.g., genetic predisposition, emotional neglect, or cultural pressures) converge on the same diagnostic endpoint, are essential in conceptualizing the heterogeneity of ED presentations.

This paper adopts a biopsychosocial perspective to review current evidence and emerging insights related to eating disorders in children. By examining the intersecting roles of genetics, epigenetic regulation, early caregiving environments, and sociocultural stressors—particularly those amplified during the COVID-19 pandemic—we aim to provide an integrative synthesis that can inform both preventive strategies and targeted interventions. Recognizing early indicators, enhancing diagnostic accuracy, and implementing family-centered, developmentally appropriate treatments are critical steps forward in reducing the burden of EDs in pediatric populations.

Genetic factors play a critical role in the etiology of EDs, with twin studies demonstrating heritability estimates ranging from approximately 40% to 60% [12]. Genetic vulnerabilities likely interact with environmental stressors, such as those introduced by the COVID-19 pandemic, to precipitate or exacerbate EDs [13]. Recent advancements in genomic research have identified potential genetic markers and candidate genes, notably those related to the dopamine transporter gene (DAT1), implicated in emotional regulation and impulsivity, which are commonly disrupted in individuals with EDs [14,15]. Additionally, epigenetic mechanisms, including DNA methylation, have been proposed as crucial mediators in the gene–environment interplay affecting eating behaviors, emotional regulation, and stress responses [16,17]. Epigenetic modifications may offer explanatory insights into how early-life environmental stressors and parental psychopathology impact child psychopathology, potentially predisposing individuals to EDs [18,19].

Peculiar importance to the understanding of EDs in children should also be recognized in the influential role played by parental psychopathology and family interaction patterns, particularly in feeding contexts. Parents’ psychological conditions, including depression, anxiety, and EDs themselves, profoundly influence children’s developmental trajectories, impacting emotional regulation, attachment security, and vulnerability to psychopathological conditions, including EDs [20,21]. High-quality parental interactions, especially during early feeding experiences, have been identified as protective factors, fostering healthy eating behaviors and emotional regulation [22,23]. Conversely, compromised parent–child interactions, often worsened by parental psychopathology, have been consistently associated with the emergence and maintenance of disordered eating patterns in offspring [24,25].

Given these multifaceted risk factors—genetic, epigenetic, psychological, and environmental—understanding EDs in children requires an integrated biopsychosocial approach. This perspective paper synthesizes current findings on genetic and epigenetic contributions, examines the impact of the COVID-19 pandemic, explores the crucial role of parental psychopathology and interactions, and highlights recent advancements in prevention and intervention strategies. By integrating these complex factors, this comprehensive perspective aims to enhance the understanding and management of EDs in children, informing both clinical practice and future research directions.

## 2. Genetic and Epigenetic Contributions to Eating Disorders

Genetic predispositions to eating disorders have been well-established through numerous twin and family studies. Heritability estimates for anorexia nervosa, for example, have been reported to range from 48% to 74%, while bulimia nervosa and binge eating disorder show slightly lower but still substantial genetic contributions [26,27]. These findings underscore the importance of considering genetic vulnerabilities when evaluating early-onset eating disorders in children. Variants in genes related to the serotonergic and dopaminergic systems, including 5-HTTLPR for the former and DAT1 for the latter, have been repeatedly implicated in emotional regulation, reward sensitivity, and appetite control—traits closely tied to disordered eating behaviors [28,29].

We have recently investigated the association of polymorphisms of the dopamine D4 receptor (DRD4) gene and the DAT1 gene with disturbed eating in young children and assessed the quality of mother/child interaction during feeding [30].

We specifically analyzed the distribution of variable number of tandem repeat (VNTR) polymorphisms of both genes in cohorts of three-year-old children in the overeating, undereating, and control groups. We also evaluated the quality of mother–child interactions during feeding and the presence of children’s externalizing, internalizing, and dysregulation symptoms.

Our findings showed the presence of an association of both polymorphisms with children’s eating behavior, the quality of interaction with their mothers, and their psychological symptoms. Regarding the DRD4 polymorphism, we found that the 4-tandem repeat-carrying allele behaves as a protective factor and the 2-repeats and 7-repeats alleles as risk factors for undereating behavior, the general quality of mother–child interaction, and children’s symptoms. Regarding the DAT1 polymorphism, our findings revealed that the 9-repeats allele behaves as a protective factor and the 10-repeats allele as a risk factor for overeating behavior, the general quality of mother–child interaction, and children’s symptoms. We also found a gene x gene interaction between the DAT1 9-repeat or 10-repeat allele and the DRD4 4-repeat allele. Altogether, these observations suggest a role for DRD4 and DAT1 in an early susceptibility to eating disorders [30].

Beyond candidate gene studies, genome-wide association studies (GWAS) have identified specific loci, such as those on chromosome 12, associated with anorexia nervosa, suggesting a neurobiological and metabolic underpinning to the disorder [31]. These insights help frame EDs not merely as psychological issues but also as complex, systemic conditions. Importantly, these genetic markers do not act in isolation but interact dynamically with environmental exposures.

It is now well established that this gene–environment interplay acts via epigenetic mechanisms. Epigenetic changes, such as DNA methylation, can alter gene expression in response to environmental stressors without altering the underlying nucleotide sequences [32]. Several studies have identified altered methylation patterns in genes related to the hypothalamic-pituitary-adrenal (HPA) axis, emotional regulation, and appetite control in children and adolescents with eating disorders [33,34]. For instance, hypermethylation in the oxytocin receptor gene (OXTR) has been linked to emotional dysregulation and insecure attachment, both of which are known risk factors for EDs [35].

Importantly, maternal stress and psychopathology during pregnancy have been associated with epigenetic alterations in offspring, influencing their stress responses and emotional development [36]. This transgenerational transmission of risk suggests that children exposed to maternal eating disorders or depression may carry epigenetic markers predisposing them to psychopathology. This phenomenon is particularly salient in light of evidence showing increased psychiatric symptoms in children born to mothers with binge eating disorder or anorexia nervosa [37,38].

Together, these genetic and epigenetic findings point to the necessity of considering biological predispositions in both research and clinical interventions. This biopsychosocial model recognizes that while genes load the gun, it is the environment that pulls the trigger—a framework that is essential when designing early prevention and intervention strategies for children at risk of eating disorders.

While these findings offer promising insights into the biological underpinnings of early eating behaviors, their generalizability remains limited by several factors. Many candidate gene studies, including those on DRD4 and DAT1 polymorphisms, have been conducted on relatively small, homogeneous samples, which may restrict their applicability across broader populations [39]. Moreover, gene–environment interactions are inherently complex and context-dependent, requiring replication across diverse cultural, socioeconomic, and developmental settings to validate observed associations. Similarly, although epigenetic mechanisms provide a compelling explanatory model, current evidence is still emerging, and methodological variability—such as tissue specificity and timing of sample collection—poses challenges to consistency and interpretation. A more nuanced, critical appraisal of these findings is essential to avoid deterministic interpretations and to guide future research toward more ecologically valid and replicable designs [40].

## 3. Impact of the COVID-19 Pandemic

The COVID-19 pandemic has had unprecedented effects on global health, with a particularly profound impact on the mental health of children and adolescents. Confinement measures, closure of schools, and the transition to remote learning disrupted the daily structure essential for children’s psychological well-being and routine behaviors. Several longitudinal studies have reported an increase in emotional and behavioral difficulties in children during the pandemic, particularly highlighting the exacerbation of eating-related pathologies [41,42].

Disruptions in meal patterns, increased screen time, and reduced physical activity created conditions that both reinforced and initiated disordered eating behaviors. Adolescents reported greater preoccupation with body image, influenced by increased exposure to idealized images on social media and the absence of peer interaction that typically serves as a moderating influence [43]. Emotional eating, characterized by using food as a coping mechanism for anxiety or boredom, became more prevalent, especially in families experiencing high levels of COVID-related stress [44].

Beyond disruptions in routine and emotional stability, the reduction in physical activity during the pandemic represented a significant threat to children’s physical and mental health. Regular movement is not only crucial for metabolic balance but also plays a key role in stress regulation, emotional well-being, and healthy eating behaviors [45]. The absence of structured physical activities and outdoor play contributed to increased sedentary behaviors, emotional dysregulation, and heightened body image concerns. Moreover, parental influences remained central: eating patterns modeled by caregivers, parental attitudes toward performance and perfectionism, and task-related pressure may have exacerbated children’s vulnerability to disordered eating, particularly in high-stress family environments [46].

Family dynamics also played a central role. The pandemic heightened stress within households, particularly in families already facing financial insecurity or psychological distress. Parents experiencing anxiety, depression, or disordered eating themselves often displayed disrupted feeding interactions and emotional availability, contributing to the deterioration of children’s eating behaviors [47]. In households where both parents exhibited psychological difficulties, studies observed a compounding effect on child psychopathology, with a sharper rise in internalizing symptoms such as anxiety and withdrawal, as well as externalizing behaviors including oppositionality and impulsivity [48].

Neurobiologically, the chronic stress associated with the pandemic may have affected cortisol regulation and the HPA axis functioning in children, with downstream effects on appetite and emotional regulation [49]. This adds a physiological dimension to the observed behavioral patterns, supporting the notion that EDs in children are multifactorial, with biological, psychological, and environmental contributors. Overall, the pandemic served as a real-time stress test that illuminated pre-existing vulnerabilities while generating new ones, reinforcing the need for adaptable, evidence-based mental health frameworks to address EDs in early life.

## 4. Parental Psychopathology and Interaction Patterns

Parental psychopathology has long been recognized as a critical environmental influence on child development, particularly in the domain of eating behaviors and emotion regulation. When a parent—especially a primary caregiver—struggles with psychological disorders such as depression, anxiety, obsessive-compulsive disorder (OCD), or an eating disorder themselves, the parent–child relationship often suffers in ways that contribute to the emergence of disordered eating in children [50]. These disorders impair the parent’s ability to engage in sensitive, consistent, and emotionally responsive caregiving, particularly during routine contexts such as feeding interactions. As feeding is among the earliest and most recurrent relational rituals in infancy and early childhood, disturbances in this context may have long-lasting repercussions on how children understand hunger, satiety, emotional states, and interpersonal trust [51,52].

Several studies have found a strong association between maternal psychopathology and childhood internalizing and externalizing symptoms, including emotional withdrawal, anxiety, aggression, and impulsivity—all of which are behavioral profiles closely linked with the risk of developing an eating disorder later in life [53,54]. Specifically, children of mothers with eating disorders often display more problematic eating behaviors, elevated emotional dysregulation, and difficulties in forming secure attachments, especially when maternal psychopathology is present during critical developmental periods [55]. Moreover, the literature has identified that fathers, although less frequently studied, also contribute significantly to child outcomes. Paternal depression, for example, has been associated with increased oppositionality and affective dysregulation in children, suggesting that both parents’ psychological functioning should be considered in ED research and clinical assessment [56,57].

Despite increasing recognition of paternal contributions to child development, the existing literature remains disproportionately focused on maternal psychopathology. This imbalance represents a significant limitation, as it may obscure important paternal influences on the onset and maintenance of disordered eating behaviors in children. Given that paternal mental health—particularly depression, anxiety, and eating-related concerns—has been linked to adverse developmental outcomes, future research should prioritize a more balanced exploration of both parents’ roles [58]. Expanding the evidence base in this direction would not only improve etiological models but also inform more comprehensive and inclusive intervention strategies.

The emotional climate of the household and the quality of the dyadic exchanges between parent and child during feeding times serve as key predictors of future psychopathology. When caregivers are emotionally withdrawn, highly anxious, or excessively controlling, children are less likely to internalize positive feeding cues and may instead develop avoidant or anxious behaviors around food [59]. Behavioral modeling occurs through both overt and subtle parental behaviors, which children often internalize as normative. For instance, verbalized food anxieties—such as parents expressing guilt over eating certain foods or openly labeling foods as “good” or “bad”—can shape restrictive attitudes in children from an early age [60]. Similarly, frequent comments about weight, dieting, or dissatisfaction with one’s body can promote internalized body image concerns. Even covert control strategies, such as silently monitoring a child’s food intake, limiting portion sizes without explanation, or offering food conditionally based on performance or behavior, can contribute to disordered eating patterns [61]. These modeled behaviors are particularly influential when they occur consistently during emotionally salient moments, such as mealtimes or after emotionally charged events. This has been particularly well-documented in studies utilizing video-based observational methodologies, such as the SVIA (Scala di Valutazione delle Interazioni Alimentari), which evaluate real-time interactions between mothers and children during feeding. These analyses reveal that disordered maternal affect and intrusive feeding practices correlate strongly with poor child emotional regulation, refusal to eat, and excessive somatic complaints [62,63].

It is also worth considering whether the risk of developing disordered eating may be amplified in only children, who often become the exclusive focus of parental attention and expectations. In such family constellations, the intensity of dyadic interactions may heighten the child’s sensitivity to parental emotional states, caregiving inconsistencies, and implicit demands for performance or perfection [64]. Furthermore, the overall quality of parental relationships—marked by marital conflict, lack of cohesion, or inconsistent co-parenting—has been shown to significantly influence children’s emotional security and regulatory abilities. Dysfunctional parental dynamics may inadvertently shape maladaptive coping strategies in children, including disordered eating behaviors, as a response to emotional insecurity or as an attempt to exert control [65].

Further, intergenerational transmission of eating disorder symptoms has been hypothesized to occur not only through behavioral modeling and emotional contagion but also via epigenetic changes induced by maternal stress. Maternal psychopathology during pregnancy and early childhood may lead to biological alterations in the offspring that heighten sensitivity to stress and reduce coping mechanisms, thereby increasing the risk of internalizing disorders and EDs [66]. In homes where both parents are affected, these risks appear to compound, creating a cumulative vulnerability environment for the child.

Importantly, the interactions in families affected by psychopathology are not merely deficient in positive reinforcement but are often saturated with misattuned emotional signals. Such environments can inhibit the development of emotional literacy and resilience in children. Emotional dialogues—such as those studied in mother–child narrative exchanges—are typically less coherent, less emotionally supportive, and more fragmented in families where maternal anorexia, depression, or anxiety is present [67]. These findings underline the importance of targeting family communication and emotion regulation in preventive and therapeutic interventions.

## 5. Intervention

In light of these findings, clinical interventions should prioritize comprehensive family assessments, targeting both maternal and paternal psychological functioning, and the quality of their relational dynamics. Therapies that incorporate elements of attachment-based work, emotional coaching, and mindful feeding practices are especially promising in reshaping the parent–child dyad and mitigating the risk of EDs in vulnerable children.

Given the multifactorial nature of eating disorders in children, encompassing genetic, environmental, and psychological dimensions, effective prevention and intervention strategies must integrate comprehensive, multidisciplinary approaches. Early identification and intervention remain pivotal, as early-stage treatment significantly improves prognosis and reduces long-term psychological and physical health consequences [68].

Early detection of risk factors and prodromal symptoms plays a critical role in preventing the onset or worsening of EDs. Screening tools such as the SCOFF questionnaire and the Eating Attitudes Test (EAT-26) have demonstrated effectiveness in identifying early symptoms among adolescents [69,70]. Recent guidelines suggest routine implementation of these instruments in pediatric and educational settings to facilitate timely referrals [71]. Teachers and school counselors, when trained to recognize subtle cues of disordered eating or emotional withdrawal, can play an integral role in early detection.

Given their central role in shaping children’s behavioral and emotional development, parents bear primary responsibility for modeling and reinforcing healthy eating patterns, emotional regulation strategies, and attitudes toward body image [72]. Their active involvement is essential not only in clinical interventions but also in preventive efforts across developmental stages. Collaborative engagement between parents and educators, starting from early childhood education, is crucial for reinforcing consistent health-promoting messages and identifying early warning signs of disordered eating [73]. This partnership can create a cohesive support system that bridges home and school environments, enhancing the effectiveness of screening, education, and intervention efforts.

Considering the significance of the familial context, family-based interventions (FBIs) have emerged as some of the most effective treatments for pediatric eating disorders. The Maudsley Approach, a well-documented form of FBT, positions parents as central agents in the refeeding and behavioral recovery process [74]. Evidence indicates that FBT is associated with higher rates of remission compared to individual therapy for adolescents with anorexia nervosa [75]. Interventions also focus on restoring healthy feeding dynamics and reducing parental criticism or overcontrol, which can exacerbate symptoms [76].

The COVID-19 pandemic catalyzed the expansion of digital mental health services. Online platforms for cognitive-behavioral therapy (CBT) and guided self-help models have demonstrated efficacy in reducing disordered eating behaviors in both children and adolescents [77]. Furthermore, video-feedback interventions targeting parent–child feeding interactions, such as those developed by Cimino and colleagues, have shown promise in enhancing maternal sensitivity, improving child regulation, and decreasing feeding-related conflict [78]. These digital tools are particularly valuable for reaching families in remote or underserved regions.

Psychoeducational initiatives tailored for children and their caregivers aim to build knowledge about nutrition, body image, and emotional regulation. These programs are most effective when culturally sensitive and embedded within the child’s daily environments, such as schools [79]. Universal prevention programs, like the Body Project or Media Smart, have demonstrated success in reducing thin-ideal internalization and unhealthy dieting practices [80]. School-based curricula that include peer discussions, teacher training, and parent involvement are especially impactful in shifting attitudes and behaviors at the community level.

## 6. Integration of Biological Insights

Emerging biological insights also offer avenues for novel interventions. Genetic counseling may eventually become part of family education for those at high familial risk. Meanwhile, the study of epigenetic markers opens the possibility of interventions targeting stress regulation systems in children, potentially through mindfulness-based or trauma-informed care approaches that aim to reverse environmentally induced epigenetic vulnerabilities [81].

Effective intervention must be multifaceted, combining emotional support, relational trust-building, and the creation of positive role models within both family and school contexts [82]. Strengthening the authority and coherence of parents and teachers in guiding children’s health behaviors is a key component, especially in early developmental stages. m [83,84]. Such comprehensive approaches ensure that children receive consistent, supportive, and health-promoting input across all major environments of development.

In conclusion, the prevention and treatment of eating disorders in children require a layered approach that addresses individual, familial, and societal factors. As the field evolves, integration of biological, psychological, and digital tools into public health strategies will be essential to reach and support at-risk populations early and effectively.

## 7. Conclusions

In recent years, the field of child and adolescent mental health has increasingly recognized the urgency of addressing eating disorders in younger populations. These disorders are no longer limited to adolescents or young adults but are presenting earlier in development, often with unique and more severe clinical characteristics. As outlined in this paper, the etiology of childhood-onset eating disorders is multifactorial, involving an intricate blend of genetic vulnerabilities, epigenetic modifications, psychosocial stressors, parental psychopathology, and disruptions in early relational and environmental contexts.

The COVID-19 pandemic served as a global catalyst, exposing and intensifying existing vulnerabilities among children and adolescents. Disruptions to daily routines, social isolation, and elevated familial stress have all contributed to an alarming rise in disordered eating behaviors. These findings emphasize the necessity for robust, adaptable, and holistic mental health frameworks that can respond to the dynamic needs of at-risk children.

Parental psychopathology and feeding interactions further underscore the relational nature of eating disorders. Disruptions in emotional attunement and caregiving consistency—often driven by maternal and paternal psychological symptoms—highlight the critical need for family-centered prevention and intervention models. Approaches that incorporate emotional regulation, attachment theory, and responsive feeding strategies are well-positioned to mitigate these intergenerational risk factors.

Equally important are the ongoing advances in genetics and epigenetics, which promise to refine our understanding of individual vulnerability and resilience. While we are still in the early stages of integrating these insights into clinical practice, the potential for personalized, biology-informed interventions is compelling. Preventive strategies must increasingly draw from interdisciplinary insights, bridging neurobiology, psychology, and family systems theory to create a more accurate and compassionate framework for diagnosis and care.

Finally, the way forward must also address systemic challenges, including limited access to specialized care, the digital divide, and the cultural stigmatization of mental illness. Expanding school-based interventions, telemedicine infrastructure, and community awareness programs are crucial steps toward equity in prevention and treatment. Addressing eating disorders in children is not only a matter of clinical relevance—it is a developmental and ethical imperative. Failure to intervene early risks compromising not only psychological well-being but also cognitive, emotional, and physical growth during critical windows of development. As the burden of childhood EDs continues to grow, so too must our commitment to innovation, early detection, and integrative care strategies. The stakes are not merely clinical—they are profoundly developmental, shaping the psychological and physical trajectories of our most vulnerable populations.

## Data Availability

Not applicable.
